# 妊娠绒毛膜癌肺转移的诊治进展

**DOI:** 10.3779/j.issn.1009-3419.2011.10.06

**Published:** 2011-10-20

**Authors:** 志庸 张

**Affiliations:** 100730 北京，中国医学科学院，北京协和医学院胸外科 Department of Thoracic Surgery, Peking Union Medical College Hospital, Chinese Academy of Medical Sciences, Beijing 100730, China

**Keywords:** 妊娠绒毛膜癌, 肺转移, 诊断与治疗, 手术指征, Gestational choriocarcinoma, Pulmonary metastasis, Diagnosis and treatment, Surgical indication

## Abstract

妊娠绒毛膜癌（简称绒癌）是最常见的妊娠滋养细胞肿瘤，极易发生血行转移，常出现肺转移。自一系列有效化疗药物用于绒癌治疗之后，绒癌已成为可治愈的恶性肿瘤之一，但耐药及复发仍是治疗失败的主要原因，特别是肺转移灶的处理。如何掌握手术指征和时机成为治疗难点。本文就绒癌肺转移的诊断、化疗、手术指征及时机、手术方式等治疗进展进行综述。

## Abstract (en)

Gestational choriocarcinoma is the most common gestational trophoblastic neoplasia. It is prone to hematogenous metastasis, most commonly to the lungs. With the advent and development of chemotherapy, choriocarcinoma has become a curable tumor. However, patients with drug-resistant and recurrent choriocarcinoma are difficult to treat, even with the management of pulmonary metastasis. Resorting to surgery is also a tough decision given the challenges of identifying the appropriate surgical indication and timing. This review discusses the basic principles of management as well as recent advances in the diagnosis and treatment of patients with pulmonary metastasis of gestational choriocarcinoma.

妊娠绒毛膜癌（简称绒癌）（gestational choriocarcinoma），是最常见的妊娠滋养细胞肿瘤（gestational trophoblastic neoplasia, GTN），是由细胞滋养细胞恶性转化形成的高度恶性肿瘤，极易发生血行转移，常出现肺转移。多继发于葡萄胎，也可继发于流产、异位妊娠及早产或足月产后。临床表现为阴道不规则出血及转移灶引起的相关症状。自一系列有效化疗药物用于绒癌治疗之后，绒癌已成为可治愈的恶性肿瘤之一，但耐药及复发仍是治疗失败的主要原因，特别是肺转移灶的处理，如何掌握手术指征和时机成为治疗难点^[1]^。

## 1 疾病特点

### 1.1 流行病学及病因

绒癌有明显的种族、地域差异，我国发生率约1/2, 882次妊娠，属高发区域^[2]^。欧洲及北美约1/40, 000次妊娠，东南亚和日本分别为9.2/40, 000次和3.3/40, 000次妊娠。病因可能与遗传因素有关，危险因素包括完全性葡萄胎史、高龄产妇、种族等^[3]^。绒癌肺转移发生率达85.1%^[1]^。

### 1.2 肿瘤生长方式及生物学特点

绒癌的特点是滋养干细胞失去控制而异常增生，以致失去原有绒毛结构，侵润破坏血管的能力极强，不断浸润溶解子宫内膜基质、侵入子宫肌层，早期即侵透血管而血行转移。最常见的转移部位为肺（80%）、阴道（30%）、脑（10%）、肝（10%），淋巴系统转移较罕见^[4, 5]^。绒癌癌栓脱落后循静脉回流经右心进入肺动脉，可栓塞肺动脉小分支，绒癌细胞增殖侵透血管壁后，破坏肺组织，与血肿混合而成肺转移灶；侵透肺小静脉后，癌细胞经左心随体循环动脉转移到脑、肝等全身各脏器^[2, 5]^。因此肺常是血行转移的第一站，跳跃肺的远处转移极为罕见^[6]^。若未能有效控制肺转移，极易发生脑转移等远处转移，成为绒癌最常见的死亡原因。

### 1.3 病理及免疫组化特点

绒癌肺转移灶中心常为凝血块及坏死组织，外周围绕着两层恶性滋养细胞：内层为单核的细胞滋养细胞层，可有少量核异型和不典型有丝分裂像，但与预后无关；外层为多核的合体滋养细胞层，特征性缺失绒毛膜绒毛，绒毛膜促性腺激素（human chorionic gonadotropin, hCG）染色阳性^[4]^。周围肺组织受压塌陷、出血、水肿和炎性细胞浸润。免疫组化有助鉴别诊断，合体滋养细胞β-hCG强阳性，人胎盘生乳素（human placental lactogen, hPL）弱阳性^[7]^。绒癌hCG、抑制素（inhibin）、人类白细胞抗原-G（human leukocyte antigen-G, HLAG）、黑色素瘤细胞粘附分子（melanoma cell adhesion molecule, Mel-CAM, CD146）常呈阳性^[8]^。有效化疗可使肺转移灶滋养细胞死亡，残留凝血块及坏死组织机化并形成纤维包裹；化疗失败者，出血坏死区内仍散在少数滋养细胞^[1]^。有时病灶较小又无明显出血坏死，易被误认为低分化鳞癌，需结合子宫有原发绒癌病灶、血或尿hCG升高、肿瘤细胞无细胞间桥以及免疫组化hCG阳性鉴别诊断^[7]^。

### 1.4 临床表现

早期绒癌肺转移通常无明显临床表现，转移灶较多、较大时可有咯血、胸闷憋气、胸痛甚至血胸等表现，严重者可出现呼吸衰竭。目前由于绒癌患者需定期复查胸部CT，常能在早期无症状时发现肺转移灶^[2]^。

## 2 诊断

### 2.1 诊断标准

育龄女性，依据确定的绒癌病史或产后或流产后阴道持续不规则出血，血、尿β-hCG异常升高而疑诊绒癌，结合肺部CT演进出现转移阴影，可临床诊断绒癌肺转移，确诊需子宫或转移灶切除后的病理检查。绒癌细胞可分泌hCG，测定β-hCG是协助诊断、评价疗效及随诊的敏感而特异性指标^[1, 2]^。

准确评估患者应包括行盆腔超声或MRI或动脉造影，了解原发灶范围及位置，行胸腹CT、头颅MRI了解全身情况^[6, 9]^。PET-CT能有效检测出CT及MRI未能发现的隐匿转移或复发病灶^[9]^。

>60%的绒癌患者初诊时已发生肺转移。绒癌肺转移的胸部X线特点为圆形结节状^[1]^。CT典型表现为肺外周或胸膜下单发或多发类圆形软组织密度结节影。转移灶的影像演进过程中，早期可表现为小片状影或磨玻璃样微结节影，逐渐增浓、增密、增大，形成边缘不光滑的结节或肿块，少有胸膜牵拉，最终发展为边缘清晰、密度均一的结节或肿块，可有空洞，肺门或纵隔淋巴结常无肿大。化疗后肺内转移灶影像表现可呈现动态变化过程。随着绒癌肺转移灶的增大，将特征性伴随血β-hCG上升。对化疗和随诊过程中新出现的肺内阴影，要结合血β-hCG水平变化判定是否为疾病进展^[2]^。穿刺活检绒癌肺转移灶出血播散风险高，且阳性率低，既不必要也不推荐^[5]^。

### 2.2 鉴别诊断

注意与肺内其它病变相鉴别，如原发肺癌、结核瘤、化疗引起的肺组织损害及免疫力下降导致的肺部感染等^[10]^。

### 2.3 分期

目前通用国际妇产科联盟颁布的FIGO2000版GTN分期评分系统（表 1、表 2），总分≤6分为低危组，>6分为高危组^[6]^。

**1:** FIGO 2000滋养细胞肿瘤分期^[6]^ FIGO 2000 staging and classification of GTN

Stage	
Stage Ⅰ	Disease confined to the uterus
Stage Ⅱ	GTN extends outside of the uterus, but is limited to the genital structures (adnexa, vagina, broad ligament)
Stage Ⅲ	GTN extends to the lungs, with or without known genital tract involvement
Stage Ⅳ	All other metastatic sites

**2:** 评分系统 Scoring system

Factor	Score			
	0	1	2	4
Age	< 40	≥40	-	-
Antecedent pregnancy	Mole	Abortion	Term	-
Interval months from index pregnancy	< 4	4- < 7	7- < 13	≥13
Pre-treatment serum hCG (IU/mL)	< 10^3^	10^3^-10^4^	10^4^-10^5^	≥10^5^
Largest tumour size (cm) (including uterus)	< 3	3- < 5	≥5	-
Site of metastasis	Lung	Spleen, kidney	Gastro-intestinal	Liver, brain
Number of metastases	-	1-4	5-8	>8
Previous failed chemotherapy	-	-	Single drug	2 or more drugs

## 3 治疗

绒癌的治疗原则以化疗为主，辅以手术、放疗、介入等综合治疗，低危组对甲氨蝶呤（MTX）或放线菌素D（ActD）单药化疗耐药风险较低，高危组需要多药联合化疗^[6]^。绝大多数患者化疗后绒癌肺转移灶即可消失。

### 3.1 随诊及疗效判断标准

随诊患者应保证避孕条件下每周查血β-hCG，至连续3周阴性后，每月查1次至少12个月^[5]^。完全缓解（complete response, CR）指化疗后连续3次血β-hCG正常，且转移灶消失。部分缓解（partial response, PR）指化疗后β-hCG下降至初治时的50%以下，转移灶缩小。耐药指化疗2个疗程后，β-hCG下降不满意（未成对数下降），或呈平台状，甚至上升，影像提示病灶不缩小或变大，或出现新发转移灶。复发指完全缓解停止化疗3个月以上，再次出现血β-hCG升高（排除妊娠）或影像发现新发病灶^[1]^。病理阳性指病灶内滋养细胞残留或免疫组化hCG或β-hCG染色阳性^[11]^。

### 3.2 化疗

EMA/CO方案[依托泊苷（VP-16）+MTX+ActD/环磷酰胺（CTX）+长春新碱（VCR）]毒性低，缓解迅速，被公认为目前治疗高危患者的一线化疗方案。对初治化疗耐药的绒癌患者多采取更换方案的挽救性化疗，仍无效者需考虑手术切除耐药病灶^[12]^。

EMA/EP方案[VP-16+MTX+ActD/VP-16+顺铂（DDP）]是针对5-FU联合长春新碱和放线菌素或EMA/CO化疗耐药患者的有效补救方案^[13, 14]^。已转移的高危GTN患者初步EMA/CO治疗缓解率达66.7%，经含铂方案挽救性化疗、手术切除残留病灶及全脑放疗等综合治疗，完全缓解率达93.3%^[15]^。紫杉醇可作为三线方案，60%的高危转移患者经EMA/CO一线治疗治愈，19%经二、三线挽救性化疗及有选择的手术治疗治愈，长期缓解率为81.4%，5年生存率为78%^[16]^。

Feng等^[14]^对91例耐药和复发性GTN患者以毒性更低的FAEV方案[氟尿苷（FUdR）+ActD+VP16+VCR]化疗，完全缓解率为60.4%，辅以手术治疗后，3年生存率为74.9%。适时合理地实施辅助手术，能够提高复发耐药患者的缓解率^[12, 14]^。

### 3.3 手术指征及时机

90%以上的绒癌肺转移患者，经规范化疗即可取得完全缓解效果。仅有少数具有肺部耐药病灶或复发相关病灶患者需要手术治疗，只有手术指征选择适当才能真正发挥治疗作用。

1965年Thomford等^[17]^提出切除肺转移灶的手术指征：①患者身体状况能够耐受开胸手术；②原发灶已得到控制（已行子宫切除或动脉造影提示盆腔无肿瘤）；③全身无其它转移灶；④影像显示肺转移病灶局限于单侧肺；⑤血β-hCG水平 < 1, 000 mIU/mL。在此基础上，随着化学治疗学、病理学以及妇科学的不断进步和临床经验的积累，绒癌肺转移的手术指征也在不断修正。

合体滋养细胞易被化疗杀灭，绝大多数患者血β-hCG呈对数下降直至恢复正常。但少数化疗满意患者的肺转移灶经多疗程巩固化疗后仍存在，临床多因此行肺叶切除术，而病理检查肺内病灶多为出血坏死或纤维化，常无滋养细胞残留。此类患者的复发率并不比CR患者更高，也应视作CR而非PR^[10]^。CT所示的残留病灶虽经化疗仍难以完全吸收消失，难以确定的此类肺内病灶即为耐药转移灶，随诊患者多病情长期无进展，故可随诊观察，如血清hCG保持正常，既不应过度化疗，更不必急于手术^[2, 11]^。但必须规律随访血β-hCG及转移灶影像变化，尤其停药6个月内更要警惕复发^[10]^。

经过化疗，绒癌肺转移灶周围纤维瘫痕形成，化疗药物在病灶部位不能达到有效浓度而耐药，或化疗使瘤细胞突变而出现获得性耐药^[1]^。如经若干疗程后肺转移灶仍未完全消失，且血β-hCG下降不满意或下降曲线出现“拖尾”现象（从10 mIU/mL到2 mIU/mL下降缓慢），提示耐药滋养细胞尤其是细胞滋养细胞仍可能存活，并产生低水平β-hCG，此时建议切除肺内耐药灶、防止复发^[18]^。FIGO评分高危及达CR后巩固化疗不足2个疗程是绒癌复发的明确相关因素。复发患者使用单纯化疗获得完全缓解后的再次复发率明显高于化疗结合手术切除耐药残留病灶者，完全缓解率也明显低于化疗结合手术者^[19]^。若有证据提示肺转移灶与复发或耐药相关且病灶局限，则挽救性化疗辅以手术切除肺转移灶是提高缓解率、改善预后的有效手段。复发及耐药患者的完全缓解率分别为88.9%和78.6%，病理阳性率分别为55.5%和35.7%^[11]^。

绒癌耐药及复发患者对化疗常不敏感，手术对去除耐药病灶、防止复发有不可替代的作用。但围手术期化疗对控制可能的手术刺激瘤细胞血行转移效果不确定，故手术成功的前提是病灶稳定而局限^[11]^。术前血β-hCG水平越高，提示滋养细胞增生活跃，手术难以根除病灶，发生远处转移的几率高，预后差。手术时机应选择在血β-hCG控制正常或接近正常时施行^[11, 18]^。术前血β-hCG < 20 mIU/mL的患者均疗效良好，β-hCG>63 mIU/mL的患者均死亡。建议控制在术前血β-hCG < 10 mIU/mL时施行手术，较为安全^[1, 2]^。手术决策时必须注意全面考虑患者的全身状况，如除肺以外，其它部位存在无法根除的病灶，即使肺部病灶能够切除，也不应手术。

综上，目前的手术指征为：肺转移灶局限于一叶肺的耐药病灶，子宫原发肿瘤已有效控制，全身其它部位无转移灶，经化疗血β-hCG控制正常或接近正常水平。

### 3.4 手术技巧及围手术期处理

对局限于单一肺叶内的病灶，多行解剖性肺叶切除。由于绒癌细胞极易发生血行扩散转移，术中必须操作轻柔，避免粗暴挤压肿瘤，严格按照先结扎肺静脉后结扎肺动脉原则进行，以免肿瘤扩散。出现肺门及纵隔淋巴结转移者极少见。同侧但不同肺叶的转移灶以切除最大病灶所在一肺叶为主，其余病灶行楔形切除。肺转移灶切除后，肺内又出现新的耐药转移灶时，如仍符合手术指征，可考虑再次手术切除^[2]^。对肺周边小的单个转移灶，肺楔形切除术能够高选择性地切除肺转移灶达到治疗目的，并最大限度保留健康肺组织^[20]^。随着胸部微创外科技术的进步，已逐渐实践应用胸腔镜（video-assisted thoracic surgery, VATS）治疗绒癌肺转移。

与其它肿瘤的手术时机通常宜在化疗间歇期进行截然不同，绒癌手术治疗必须在围手术期联合化疗中进行，以减少手术造成的肿瘤播散，减灭隐匿转移灶。术后待β-hCG正常后，巩固化疗2个疗程后可考虑停药^[12]^。术后拔除胸腔引流管前，向胸腔内灌注氟尿嘧啶1, 000 mg；术前术后行腰椎穿刺，鞘内注射甲氨蝶呤可预防和治疗脑转移^[1, 2]^。

### 3.5 其它治疗与展望

放疗对2 cm以内的绒癌肺转移灶有效，但放疗后肺纤维化将使未杀死的瘤细胞更难处理而较少采用。放疗多用于局部控制绒癌脑转移或肝转移灶，减少颅内出血等并发症^[1, 20]^。

*IL-2*基因转导和*TNF-α*基因转导能够逆转绒癌细胞的耐药性^[1]^。绒癌是高度恶性上皮性肿瘤，高度表达EGFR，分子靶向治疗能针对性抑制消除原发及转移的绒癌细胞，较化疗毒性低。包括靶向EGFR激酶抑制剂（西妥昔单抗、厄洛替尼、吉非替尼），靶向Erb2受体的重组抗体曲妥珠单抗以及阻断*c-myc*癌基因表达类药物。组织基质金属蛋白酶抑制剂（tissue inhibitor of matrix metalloproteinases, TIMP）马立马司他（marimastat）可抑制肿瘤生长及转移，阻止新生血管形成。尚有以抗hCG抗体进行免疫治疗及诱导细胞凋亡的基因治疗。以上多种新疗法可能在治疗化疗耐药绒癌方面出现突破性进展^[21]^。

## 4 预后影响因素

绒癌肺转移患者预后影响因素中，末次妊娠性质、分期评分及肺转移灶部位均非影响预后的独立高危因素^[18]^。年龄>35岁及术前β-hCG>10 mIU/mL是手术疗效不佳的独立预测因素^[12]^。术前已更换4种以上化疗方案或>13个疗程的患者，手术疗效差^[11]^。术后1周-2周内血β-hCG迅速下降至正常者，手术疗效好^[22]^。

## 5 结语

在规范化疗的基础上，遵循个体化的原则，正确把握手术指征、时机、方法、切除耐药性及与复发相关的绒癌肺转移灶，是提高绒癌肺转移患者完全缓解率、降低复发率的重要手段，可明显改善绒癌肺转移患者预后。分子靶向、免疫、基因治疗等将成为新的治疗选择，微创胸外科治疗绒癌肺转移正在实践之中。
